# S100A2 Is a Prognostic Biomarker Involved in Immune Infiltration and Predict Immunotherapy Response in Pancreatic Cancer

**DOI:** 10.3389/fimmu.2021.758004

**Published:** 2021-11-23

**Authors:** Yuan Chen, Chengcheng Wang, Jianlu Song, Ruiyuan Xu, Rexiati Ruze, Yupei Zhao

**Affiliations:** Department of General Surgery, State Key Laboratory of Complex Severe and Rare Diseases, Peking Union Medical College Hospital, Chinese Academy of Medical Sciences, Peking Union Medical College, Beijing, China

**Keywords:** S100A2, tumor microenvironment, immune cells, prognostic model, immunotherapy, PD-L1, pancreatic cancer

## Abstract

Pancreatic cancer (PC) is a highly fatal and aggressive disease with its incidence and mortality quite discouraging. It is of great significance to construct an effective prognostic signature of PC and find the novel biomarker for the optimization of the clinical decision-making. Due to the crucial role of immunity in tumor development, a prognostic model based on nine immune-related genes was constructed, which was proved to be effective in The Cancer Genome Atlas (TCGA) training set, TCGA testing set, TCGA entire set, GSE78229 set, and GSE62452 set. Furthermore, S100A2 (S100 Calcium Binding Protein A2) was identified as the gene occupying the most paramount position in risk model. Gene set enrichment analysis (GSEA), ESTIMATE and CIBERSORT algorithm revealed that S100A2 was closely associated with the immune status in PC microenvironment, mainly related to lower proportion of CD8+T cells and activated NK cells and higher proportion of M0 macrophages. Meanwhile, patients with high S100A2 expression might get more benefit from immunotherapy according to immunophenoscore algorithm. Afterwards, our independent cohort was also used to demonstrate S100A2 was an unfavorable marker of PC, as well as its remarkably positive correlation with the expression of PD-L1. In conclusion, our results demonstrate S100A2 might be responsible for the preservation of immune-suppressive status in PC microenvironment, which was identified with significant potentiality in predicting prognosis and immunotherapy response in PC patients.

## Introduction

Pancreatic cancer (PC) is one of the most aggressive malignancies, with a five-year survival rate of only 10% in the United States (1). According to the latest epidemiological data, there are 495,773 new cases and 466,003 deaths of PC worldwide in 2020, making ratio of incidence and mortality close to 1:1 (2). In addition to the lack of sensitive screening methods and the rapid progression of PC, the dismal prognosis of this disease is largely attributable to the lack of valid risk prediction models and biomarkers in PC development (3). Therefore, it is of great significance to construct an effective prognostic signature of PC and find the novel biomarker for the optimization of the clinical decision-making.

Tumor microenvironment (TME), a concept developed from Paget’s “seed and soil” theory, is regarded as both a cause and consequence of tumorigenesis, which is demonstrated to provide a permissive environment for tumor initiation and progression (4, 5). In addition to fibroblasts endothelial cells, stromal cells, blood vessels and secreted factors, the TME comprises innate and adaptive immune cells, which have a profound impact on tumor development (6, 7). In recent years, the vital role of immune cells in the occurrence and progression of PC is gradually revealed (8–10). For example, Yamamoto et al. identified NBR1-mediated selective macroautophagy/autophagy of MHC-I hindered cancer cell recognition and clearance by CD8+ T cells in PC (11), and granulin secretion by metastasis-associated macrophages activates resident hepatic stellate cells into myofibroblasts, resulting in a fibrotic microenvironment that sustains metastatic PC growth (12). Meanwhile, although immunotherapy is almost ineffective for PC (13, 14), PC patients who exhibited high effector T-cell infiltration in tumor had longer overall survival (15, 16), implying that valuing immune heterogeneity and remodeling the immune microenvironment may hold promise for PC treatment. Therefore, we considered a prognostic model based on immune-related genes (IRGs) to better predict the prognosis of PC patients and optimize the clinical decision-making. Furthermore, the most paramount gene and its potential mechanisms were further explored, as well as its ability to predict patients’ response to immunotherapy.

In the present study, we constructed a prognostic model based on nine IRGs and the corresponding nomogram, which were proved to be an independent risk factor and was validated in the training set, testing set, entire set, GSE78229 set and GSE62452 set. S100A2 (S100 Calcium Binding Protein A2), a highly conserved elongation factor (EF)-hand calcium-binding protein, was identified as the gene occupying the most paramount position in the risk signature. GSEA, ESTIMATE and CIBERSORT algorithm revealed that S100A2 was closely associated with the immune status in the PC microenvironment, mainly related to lower proportion of CD8+T cells and activated NK cells and higher proportion of M0 macrophages. Meanwhile, the results of immunophenoscore (IPS) algorithm proved that patients with high S100A2 expression might get more benefit from immunotherapy. Afterwards, our own independent cohort (PUMCH cohort) was also utilized to demonstrate S100A2 was an unfavorable marker of PC, as well as its remarkably positive correlation with the expression of PD-L1.

## Materials and Methods

### Datasets Sources and Processing

Immune-related genes were extracted and integrated from the ImmPort database (https://immport.niaid.nih.gov; ≤March 1, 2021) (17). Gene expression profile, clinical information, and mutation profile of the patients were downloaded from The Cancer Genome Atlas (TCGA) dataset (https://portal.gdc.cancer.gov/; ≤March 1, 2021). Samples with inadequate clinical information and follow-up period less than 30 days were excluded. Finally, 166 cases with corresponding gene expression profiles and clinical information were included in the study (**Table 1**, detailed in **Table S1**). Gene IDs was converted to gene symbol using a GFF3 file, which was downloaded from GENCODE (https://www.gencodegenes.org/). The gene expression data was converted to TPM (Transcripts Per Kilobase Million), and log2(TPM + 0.01) was used throughout the analysis unless otherwise noted. The samples of tumor tissues in TCGA set were randomly divided into to a training set and a testing set by a ratio of 7:3 using “sample” function of R software.

**Table 1:** Clinical and pathologic information of training set, testing set and entire set.

Character	TRAINING SET		TESTING SET		ENTIRE SET	
	Number	%	Number	%	Number	%
**Age**						
**Median**	65		64.5		65	
**Range**	35–85		39–88		35–88	
**OS (M)**						
**Median**	15.3		16.1		15.6	
**Range**	1.1–72.7		1.0–91.4		1.0–91.4	
**STATUS**						
**ALIVE**	52	44.83	24	48.00	76	45.78
**DEAD**	64	55.17	26	52.00	90	54.22
**gender**						
**Male**	66	56.90	24	48.00	90	54.22
**Female**	50	43.10	26	52.00	76	45.78
**AJCC_stage**						
**I**	14	12.07	4	8.00	18	10.84
**II**	97	83.62	44	88.00	141	84.94
**III**	2	1.72	1	2.00	3	1.81
**IV**	3	2.59	1	2.00	4	2.41
**Grade**						
**G1**	17	14.66	9	18.00	26	15.66
**G2**	65	56.03	26	52.00	91	54.82
**G3**	32	27.59	15	30.00	47	28.31
**G4**	2	1.72	0	0.00	2	1.21
**T STAGE**						
**T1**	3	2.59	3	6.00	6	3.61
**T2**	16	13.79	5	10.00	21	12.65
**T3**	95	81.90	41	82.00	136	81.93
**T4**	2	1.72	1	2.00	3	1.81
**N STAGE**						
**N0**	32	27.59	13	26.00	45	27.11
**N1**	81	69.82	37	74.00	118	71.08
**NX**	3	2.59	0	0.00	3	1.81
**M STAGE**						
**M0**	59	50.86	17	34.00	76	45.78
**M1**	3	2.59	1	2.00	4	2.41
**MX**	54	46.55	32	64.00	86	51.81

Meanwhile, GSE15471, GSE28735, GSE62165, GSE62452, GSE78229, and GSE71729 dataset were downloaded from the Gene Expression Omnibus (GEO) (http://www.ncbi.nlm.nih.gov/geo/) (18–23), in which GSE62452 and GSE78229 with corresponding clinical information were used for external validation (**Table 2**, detailed in **Tables S2**, **S3**). Expression values were calculated using the robust multi-array average (RMA) algorithm except GSE71729. The normalized expression matrix of microarray data can be directly download from the GEO dataset. They were performed on GPL570, GPL6244, GPL13667, and GPL20769 platform. Probes were matched to the gene symbols using the annotation files provided by the manufacturer.

**Table 2:** Clinical and pathologic information of GSE62452 and GSE78229 dataset.

Character	GSE62452 (N = 66)		GSE78229 (N = 49)	
	Number	%	Number	%
**OS (M)**				
**Median**	14.6		14.2	
**Range**	0.9–70.8		0.9–70.8	
**STATUS**				
**Alive**	16	24.24	14	28.57
**DEAD**	50	75.76	35	71.43
**AJCC_stage**				
**I**	4	6.06	4	8.16
**II**	45	68.18	44	89.80
**III**	11	16.67	1	2.04
**IV**	6	9.09	0	0
**Grade**				
**G1**	2	3.03	2	4.08
**G2**	32	48.48	24	48.98
**G3**	30	45.45	21	42.86
**G4**	1	1.52	1	2.04
**GX**	1	1.52	1	2.04

Furthermore, a single-cell dataset CRA001160 was analyzed through Tumor Immune Single-cell Hub (TISCH) database (http://tisch.comp-genomics.org/) and Seurat package, and also cell type clustering and gene expression location analysis (24, 25). The expression profile of 51 pancreatic cell lines was integrated from the CCLE database (https://portals.broadinstitute.org/ccle) (26).

### Construction and Validation of a Risk Signature Associated With Survival of PC Patients

Limma package was applied to screen differentially expressed genes (DEGs) in GSE15471, GSE28735, and GSE62165 datasets respectively (27). |Fold Change| >1.5 and false discovery rate (FDR) <0.05 were set as the cutoffs for the DEGs. The intersection of DEGs were selected as candidate genes. Univariate Cox regression was used to identify genes that were significantly associated with overall survival (OS) of PC patients in the training set (P <0.01). Subsequently, Least absolute shrinkage and selection operator (LASSO) regression analysis was further used to screen out the optimal gene combination for constructing the risk signature. According to the regression coefficient-weighted pseudogene expression, the risk signature was established as follows: Risk score = (expr_gene1_ × Coef_gene1_) + (expr_gene2_ × Coef_gene2_) + … + (expr_genen_ × Coef_genen_). The efficiency and independence of the risk signature were assessed by Kaplan–Meier (K–M) curve, time-dependent receiver operating characteristic (ROC) curve and survival point diagram in both the internal validation set (training set, testing set, and entire set) and the external validation set (GSE78229 set and GSE62452 set). Copy number variation information of the nine genes was extracted from the cBioportal database (http://www.cbioportal.org/) (28), and protein expression in normal and tumor tissues was obtained from the Human Protein Atlas (HPA) database (https://www.proteinatlas.org/).

Meanwhile, in order to make the prediction model more accurate, the clinicopathological information was also incorporated with the riskscore to establish a nomogram, which was based on the results of the univariate and multivariate analysis by using the ‘rms’ package in R language. The C-index, calibration curve and time-dependent ROC curve of 1-, 1.5-, and 2-year were applied to evaluate the predictive effectiveness of the nomogram.

### Differential Gene Analysis, Co-Expression Network Construction and Functional Enrichments Analysis Between S100A2 High and Low Expression Group

The pan-cancer expression analysis of S100A2 was performed through the GEPIA2 database (29). edgeR package was used to perform DEGs analysis between S100A2 high and low expression group, in which |Log2FC| >2 and FDR <0.001 were considered statistically significant (30). The pheatmap package, tidyverse package, and ggrepel package were utilized to create the heatmap and the volcano plot in R language. Approximately 50 genes with the most significant differences were shown in the heatmap, and those genes with their P values <1 × 10^–20^ and |logFC| >4 were labeled in the volcano plot. Afterwards, the co-expression network was constructed and visualized with STRING database and Cytoscape. The minimum required interaction score was set to be high confidence (0.700) and disconnected nodes were hidden in the network, therefore not all genes were represented. To further elucidate the mechanism of S100A2 in the development of PC, we performed GSEA analysis of the DEGs (31). The ALL ontology of the DEGs was analyzed by Gene Ontology (GO) (32), while pathway enrichment was analyzed by the Kyoto Encyclopedia of Genes and Genomes (KEGG) (33). The number of random sample permutations was set at 1,000, and NOM p-value <0.05 and FDR q-value <0.25 were set as the significance threshold.

### Estimation of Tumor Infiltrating Immune Cells

CIBERSORT algorithm could calculate the ratios of infiltrating immune cells from tissue transcriptional profiles by a deconvolution algorithm (34). Based on the expression profiles of patients in the TCGA and GSE71729 datasets, we calculated the relative abundance of 22 types of tumor infiltrating immune cells in each patient. Meanwhile, stromal, immune, and estimate scores were outputted respectively by the R package ‘estimate’ (35).

### Tumor Mutation Burden Analysis

The mutation profile was acquired from TCGA data portal (https://portal.gdc.cancer.gov/; ≤March 1, 2021). Somatic variants data of patients were analyzed and visualized by maftools package in R language (36). Then the tumor mutation burden (TMB) of each patient was calculated and analyzed by TCGA mutations package.

### Prediction of the Patients’ Response to Immunotherapy

Immunophenoscore (IPS) was a scoring scheme for the quantification of tumor immunogenicity, which was verified to positively correlated to the probability to respond to immunotherapy (37). The Cancer Immunome Atlas (https://tcia.at/) characterized the intratumoral immune landscapes and the cancer antigenomes from 20 solid cancers (37). The IPS data of PC patients was extracted for the following analysis, including the scores for anti-PD-1/PD-L1 treatment and anti-CTLA-4 treatment. Meanwhile, the correlation between S100A2 and immune checkpoints was also investigated in TCGA entire set, including PD-1, PD-L1, and CTLA-4.

### Clinical Specimens

A total of 65 patients with primary PDAC who underwent surgical resection at the Peking Union Medical College Hospital (PUMCH) were included in this study (PUMCH cohort, April 2019–November 2020). TNM staging was evaluated according to the 8th edition of the American Joint Committee on Cancer (AJCC) staging system for PC (38). Sequential sections of each patient were used for following studies. Written informed consent were obtained from all the patients enrolled in this study. This project was approved by the Ethics Committee of Peking Union Medical College Hospital.

### Cell Culture

All pancreatic cancer cell lines were purchased from the American Type Culture Collection (ATCC). All the cell lines were tested for mycoplasma every two months and identified by STR (Short Tandem Repeat) identification. HPNE, PANC-1, T3M4 and MIACaPa-2 cell lines were cultured in high glucose Dulbecco’s modified Eagle’s medium (DMEM; CORNING, Manassas, USA), BxPC-3, AsPC-1, SW1990, PATU 8988 cell lines were cultured in RPMI-1640 medium (CORNING, Manassas, USA), and Capan-1 and CFPAC-1 cell lines were cultured in Iscove’s Modified Dulbecco Medium (IMDM; CORNING, Manassas, USA). All medium was supplemented with 10% fetal bovine serum (HyClone, Logan, UT, USA). All cell lines were routinely maintained at 37°C with 5% CO2 in a humidified incubator.

### Immunohistochemistry

Manual staining was performed as the protocol previously described in this research (39). For primary antibody incubation of each patient, two sequential sections were incubated with rabbit monoclonal anti-S100A2 antibody (1:250) (Abcam, ab109494) for 1 h and rabbit monoclonal anti-PD-L1 antibody (1:200) (Abcam, ab205921) for 1 h respectively.

### RNA Isolation and RT-PCR

Total RNA was extracted from PDAC cell lines by Trizol reagent (Ambion, Life Technologies, 15596026). The cDNA was synthesized using a cDNA Reverse Transcription kit (Thermo scientific, K1622). Quantitative PCR was performed using PowerUp™ SYBR™ Green Master Mix (Applied Biosystems, A25742) in StepOnePlus™ (Applied Biosystems) according to the manufacturer’s protocols. The primer sequences were used as follows:

S100A2: Forward 5′-GCCAAGAGGGCGACAAGTT-3’,

 Reverse 5′-AGGAAAACAGCATACTCCTGGA-3’;

GAPDH: Forward 5′-GTCTCCTCTGACTTCAACAGCG-3’,

 Reverse 5’-ACCACCCTGTTGCTGTAGCCAA-3’.

All the values were normalized to GAPDH, and the 2^−ΔCt^ method was used to quantify the fold change.

### Statistical Analysis

All the statistical analyses and visualization were performed using Rstudio (version 4.1.0) and GraphPad Prism 8 (version 8.0.1), including DEGs analysis, univariate and multivariate Cox regression analysis, LASSO regression analysis, correlation analysis, clinicopathological factor analysis, ROC curve analysis, and K-M survival analysis. A two-sided P <0.05 was considered as statistically significant unless otherwise noted.

## Results

### Nine Immune-Related Genes Were Screened Out For Constructing A Risk Signature

**Figure 1:**
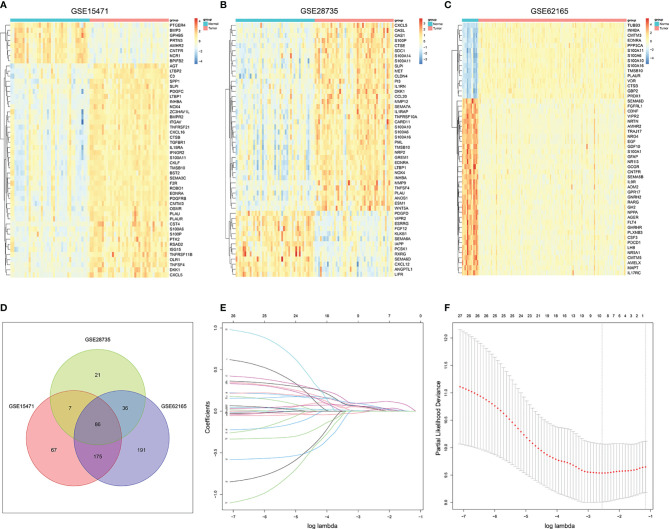
Screening out immune-related genes for constructing a risk signature. **(A–C)**. Heatmap of immune-related DEGs between PC and normal tissue in GSE15471, GSE28735, and GSE62165. **(D)** Venn plot of the intersection of three DEGs dataset. **(E)** LASSO coefficient profiles of 27 prognostic IRGs. **(F)** Cross-validation for tuning parameter selection in the LASSO model.

The flowchart of the whole analysis was illustrated in **Figure S1**. A total of 1,793 IRGs were integrated from the ImmPort database (**Table S4**) 17. First, DEGs of normal and tumor samples in GSE15471 (Normal = 36, Tumor = 36), GSE28735 (Normal = 45, Tumor = 45), and GSE62165 (Normal = 13, Tumor = 118) datasets were analyzed by limma package (|Fold Change| >1.5 and P <0.05 were considered statistically significant). Approximately 50 genes with the most significant differences were shown in the heatmap respectively (**Figures 1A**–**C**).

Then we intersected the three differential gene sets, and finally obtained 86 common DEGs (**Figure 1D**). Subsequently, univariate Cox regression analysis of 86 candidate genes was applied in TCGA training set (n = 116) to identify prognosis-related genes (P <0.01), resulting in 26 genes with Hazard Ratio (HR) >1 and one gene with HR <1 (**Table S5**). LASSO regression analysis was further performed on the prognosis-related genes in order to avoid overfitting problems and construct the risk signature, and nine genes (*AREG*, *CXCL10*, *MET*, *OAS1*, *PI3*, *PLAU*, *S100A14*, *S100A2*, and *SPP1*) were finally screened out according to the optimal lambda value (**Figures 1E, F**, log(lambda.min) = −2.554188). At the same time, the copy number variation and the protein expression status of these nine genes were also explored through the cBioportal database and the HPA database (**Figures S2**, **S3**).

## Construction of a Risk Signature For Predicting Survival Rate of PC

Base on the expression level of nine IRGs and the regression coefficient derived from LASSO regression model, we designed a risk-score formula for PC patients’ survival prediction in training set. The risk score for each patient was calculated as follows: Risk score = (0.0356 × expression level of AREG) + (0.0651 × expression level of CXCL10) + (0.1030 × expression level of MET) + (0.0269 × expression level of OAS1) + (0.0002 × expression level of PI3) + (0.0129 × expression level of PLAU) + (0.0455 × expression level of S100A14) + (0.0519 × expression level of S100A2) + (0.0404 × expression level of SPP1). Then the patients in the training set were divided into high-risk group (n = 58) and low-risk group (n = 58) according to the median cut-off value of the risk scores.

**Figure 2:**
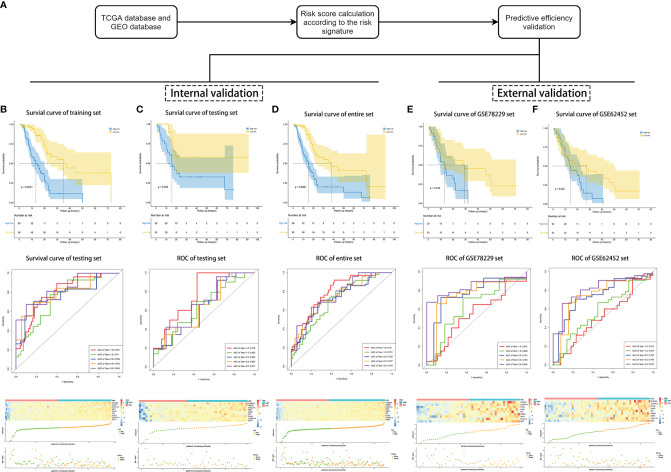
Validation of the risk signature for survival prediction in training set, testing set, entire TCGA set, GSE78229 set, and GSE62452 set. **(A)** The process of the risk signature validation. **(B–F)** Kaplan–Meier analysis of OS of the risk signature, time-dependent ROC analysis of the risk signature, heatmap of the nine hub genes expression, the risk scores distribution and survival status of the patients in training set **(B)**, testing set **(C)**, entire TCGA set **(D)**, GSE78229 set **(E)**, and GSE62452 set **(F)**.

To evaluate the competitive performance of the nine immune-related genes signature, Kaplan–Meier (K–M) curve analysis and time-dependent receiver operating characteristic (ROC) curve analysis were applied (**Figure 2A**). As shown in the Kaplan–Meier curves, patients in the high-risk group suffered worse prognosis than the patients in the low-risk group (**Figure 2B**, P <0.001). At the same time, the area under curves (AUCs) of the risk signature were 0.797 for 1 year survival, 0.740 for 1.5 year survival, 0.766 for 2 year survival, 0.794 for 2.5 year survival and 0.834 for 3 year survival (**Figure 2B**), proving a high prognostic value for survival prediction in the training set. Compared with the low-risk group, the expressions of *S100A2*, *AREG*, *CXCL10*, *MET*, *OAS1*, *PI3*, *PLAU*, *S100A14*, and *SPP1* increased in the high-risk group. Consistent with this, the number of deaths increased with the risk scores rising (**Figure 2B**).

### Effectiveness and Independence Validation of the Risk Signature for the Survival Prediction

We next performed internal validation of the risk signature in testing set (n = 50) and the entire set (n = 166), and external validation in GSE78229 dataset (n = 49) and GSE62452 dataset (n = 66). By calculating the risk scores for each patient based on the above-mentioned formula, the patients in these datasets were divided into high-risk group and low risk group using the same criteria. Consistent with the results in the training set, patients in the high-risk group had significantly lower overall survival (OS) than those in the low-risk group (**Figures 2C, D**, P <0.05). The AUCs of ROC curves for predicting 1-, 1.5-, 2-, 2.5-, and 3-year survival of PC patients in the testing set were 0.772, 0.633, 0.623, 0.634, and 0.671 respectively (**Figure 2C**), and those in the entire set were 0.790, 0.701, 0.725, 0.747, and 0.764 (**Figure 2D**). As for external validation, the AUCs of ROC curves were 0.541, 0.626, 0.761, 0.755, and 0.842 in GSE78229 dataset, and 0.512, 0.579, 0.739, 0.745, and 0.814 in GSE62452 dataset (**Figures 2E, F**). Meanwhile, the expressions of the nine hub IRGs increased significantly and the number of deaths was remarkably higher in the high-risk group, which was consistent with the results of the training set (**Figures 2E, F**).

Afterwards, we intended to investigate whether the survival prediction based on the risk signature was independent of other clinical factors (**Table 1**). Univariate Cox regression analysis and multivariate Cox regression analysis were conducted on these factors in the training set, testing set and entire set respectively. And the results showed that the risk signature was independent of other clinical factors, including age, gender, AJCC_stage, grade, T stage and N stage (**Figures S4A**–**F**, P <0.05 in all dataset for risk score). The prognostic value of the risk signature was also explored in different cohorts stratified by age, gender, tumor grade and T stage (**Figures S5A**–**L**, P <0.05 in all subgroups). Regardless of the subgroup, patients in the high-risk group suffered significantly poorer prognosis than those in the low-risk group, further confirming that this risk signature was an independent prognostic factor for PC.

### Construction and Validation of a Nomogram Based on the Nine-Gene Signature of PC

**Figure 3:**
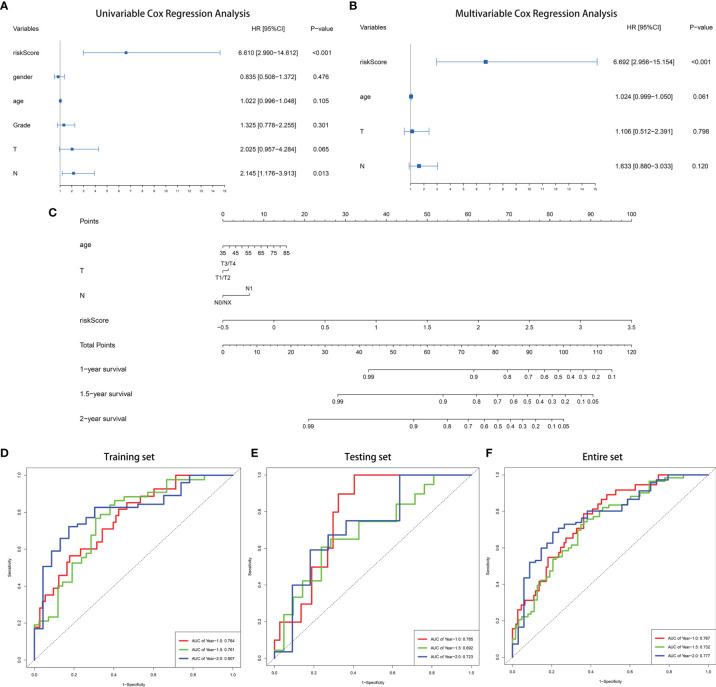
Construction of a nomogram for predicting 1-, 1.5-, and 2-year survival rate of PC. **(A)** Forrest plot of univariate Cox regression analysis in training set. **(B)** Forrest plot of multivariate Cox regression analysis in in training set. **(C)** Nomogram integrating nine IRGs-based risk score, age, T stage and N stage. **(D–F)** Time-dependent ROC analysis of the nomogram in training set, testing set and entire TCGA set.

In order to better optimize the risk signature, detailed clinical information of 166 PC patients in the TCGA dataset was collected, including age, gender, tumor grade, AJCC tumor stage and TNM stage (**Table 1**). First, we performed univariate Cox regression analyses on all the factors in training set, and then factors with P <0.2 were included in the multivariate analysis (**Figures 3A, B**). Concomitantly, we reconfirmed that risk score was an independent prognostic factor in this process. Finally, risk score, age, T stage and N stages were incorporated into the construction of nomogram for predicting 1-, 1.5-, and 2-year survival rate of PC. In the nomogram, the patients’ 1-, 1.5-, and 2-year survival rates were estimated by the total points obtained by adding up the point of each factor (**Figure 3C**). The C-index of the training set, the testing set and entire set were 0.718, 0.686, and 0.708 respectively, indicating the excellent performance of the nomogram. Subsequently, time-dependent ROC curve and calibration plot were applied to further evaluate the effectiveness of the nomogram. The AUCs of ROC curves for predicting 1-, 1.5-, and 2-year survival were 0.764, 0761, and 0.807 in the training set (**Figure 3D**), 0.785, 0.692, and 0.723 in the test set (**Figure 3E**), and 0.767, 0.732, and 0.777 in the entire set, respectively (**Figure 3F**). In addition, the calibration plot showed good agreement between the predicted and actual outcome of 1-year, 1.5-year, and 2-year OS of the nomogram in training set (**Figures S6A**–**C**), testing set (**Figures S6D**–**F**) and entire set (**Figures S6G**–**I**).

### S100A2 Is Highly Expressed and Correlates With Unfavorable Prognosis in PC

**Figure 4:**
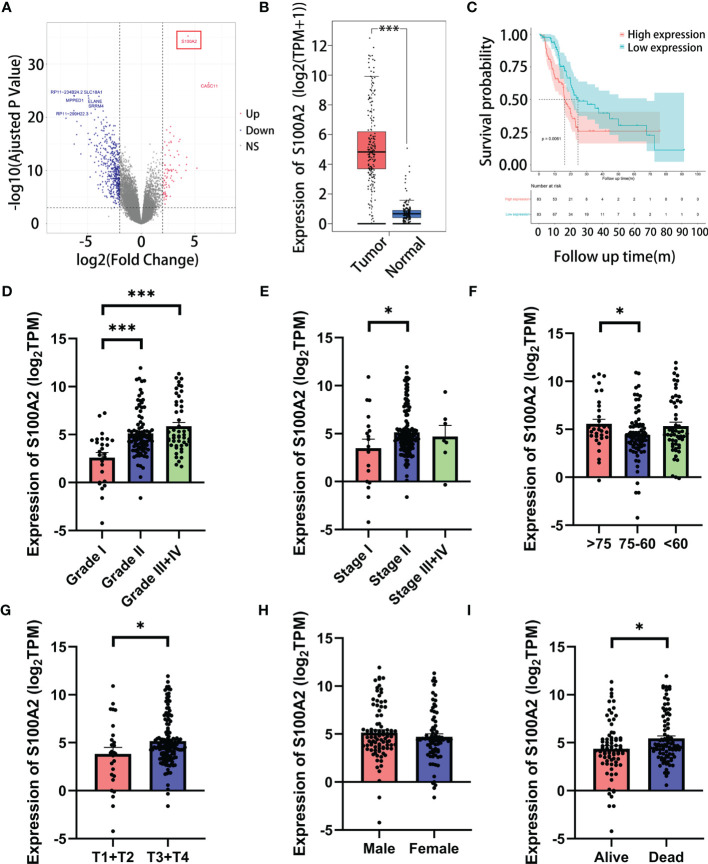
The correlation of the expression of S100A2 and clinicopathological features of PC patients in TCGA entire set. **(A)** Screening out the most paramount gene in risk signature by DEGs analysis between high and low risk groups (the gene in red box). **(B)** Expression difference of S100A2 between PC tissue and normal tissue according to the cBioPortal database. **(C)** Kaplan-Meier analysis of OS between the high S100A2 expression group and low S100A2 expression group. **(D–I) **The correlation of S100A2 expression with clinicopathological features, including grade, AJCC_stage, age, T stage, N stage and status. *P < 0.05; ***P<0.001.

In the DEGs analysis between the high and low risk groups, the increased expression of S100A2 occupied the most significant position (**Figure 4A**, FDR = 5.55 × 10^−36^, log_2_FC = 4.36). Furthermore, due to its high proportion in the risk signature, we tended to consider that S100A2 occupied the core position in the risk signature. A pan-cancer analysis of S100A2 was performed, showing that PC experienced one of the most remarkably increase of S100A2 expression among all types of cancer (**Figure S7**). To be specific, a joint analysis of TCGA and GTEx databases confirmed that the expression of S100A2 in PC tissues was significantly higher than that in normal tissues (**Figure 4B**, P <0.001). Meanwhile, TCGA entire set was divided into S100A2 high and low expression groups based on S100A2 median expression. The Kaplan–Meier analysis elucidated that PC patients with S100A2 high expression suffered a poor prognosis than those with S100A2 low expression (**Figure 4C**, P <0.01). Concomitantly, the association between S100A2 expression and patients’ clinicopathological information was further investigated. Notably, the expression of S100A2 was significantly increased along with the progression of tumor grade, AJCC_stage, age and T stage (**Figures 4D**–**I**).

**Figure 5:**
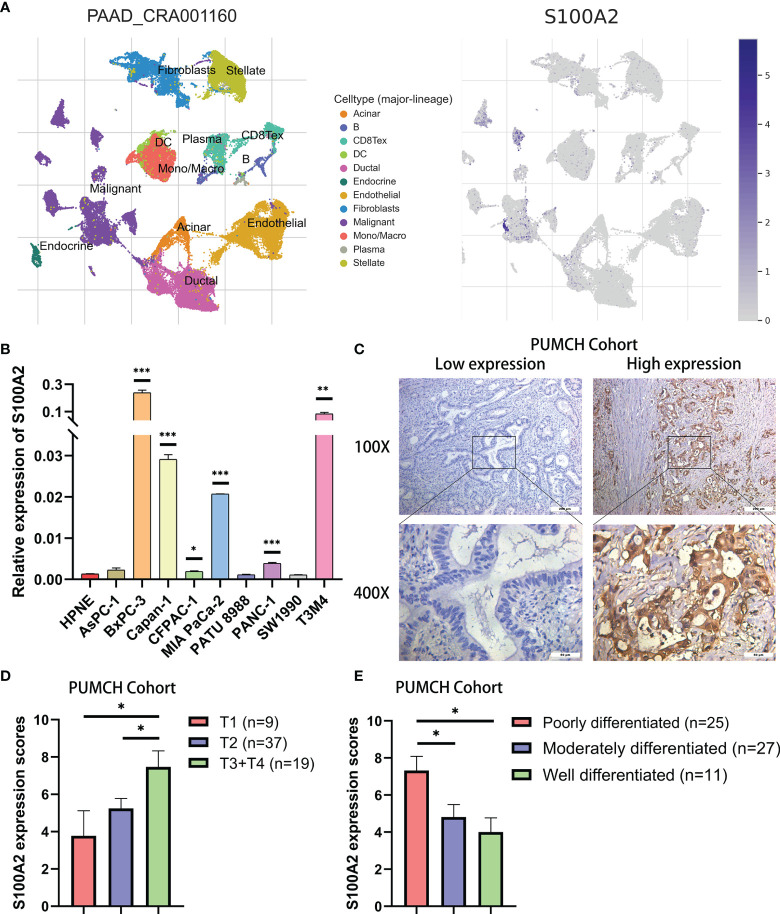
Validation of high expression of S100A2 in PC cancer cells and its association with poor prognosis. **(A)** The results of clustering and S100A2 expression distribution in single cell dataset CRA001160. **(B)** The expression difference of S100A2 between normal and pancreatic cancer cell lines detected by qRT-PCR. The difference between each PC cell line and HPNE was analyzed. **(C)** Representative images of low and high expression of S100A2 in PUMCH cohort (n = 65). **(D–E)** Correlation between S100A expression and T stage and differentiation status in PUMCH cohort. *P < 0.05; **P < 0.01; ***P < 0.001.

In order to further verify the above findings, we conducted clustering on the single-cell dataset CRA001160 and explored the predominant expression cells of S100A2 (24, 25). It was found that S100A2 was mainly expressed by cancer cells in PC tissues (**Figure 5A**). Subsequently, the significantly high expression of S100A2 in tumor cells was confirmed by qRT-PCR in pancreatic normal cell line (HPNE) and pancreatic cancer cell lines (AsPC-1, BxPC-3, Capan-1, CFPAC-1, MIA PaCa-2, PATU 8988, PANC-1, SW1990, and T3M4) (**Figure 5B**). Meanwhile, PUMCH cohort (n = 65) was utilized to further validate that high expression of S100A2 was associated with poor prognosis in PC (**Table 3**). Comprehensive analysis of S100A2 immunohistochemical scores and clinicopathologic information revealed that tumor with high S100A2 expression experienced higher T stage and poorer differentiation (**Figures 5C**–**E**). Collectively, these results indicated that high S100A2 expression in PC patients was correlated with unfavorable prognosis.

**Table 3:** Clinical and pathologic information of the PUMCH cohort.

Character	Total (n = 65)		S100A2 high expressioN (N = 34)		S100A2 low expression (n = 31)	
	Number	%	Number	%	Number	%
**Age**						
**Median**	65		64.5		65	
**Range**	38–81		40–81		38–80	
**S100A2 score**						
**Median**	6		9		3	
**Range**	0–12		6–12		0–4	
**PD-L1 SCORE**						
**Median**	8		8		4	
**Range**	1–12		2–12		1–12	
**gender**						
**Male**	28	43.08	17	50.00	11	35.48
**Female**	37	56.92	17	50.00	20	64.52
**differentiation**						
**POORLY**	25	38.46	18	52.94	7	22.58
**MODERATELY**	27	41.54	13	38.24	14	45.16
**WELL**	11	16.92	2	5.88	9	29.03
**UNknown**	2	3.08	1	2.94	1	3.23
**T stage**						
**T1**	9	13.85	2	5.88	7	22.58
**T2**	37	56.92	18	52.94	19	61.29
**T3**	17	26.15	13	38.24	4	12.90
**T4**	2	3.08	1	2.94	1	3.23
**N stage**						
**N0**	28	43.08	17	50.00	11	35.48
**N1**	30	46.15	15	44.12	15	48.39
**N2**	7	10.77	2	5.88	5	16.13
**M stage**						
**M0**	63	96.92	32	94.12	31	100.00
**M1**	2	3.08	2	5.88	0	0

### S100A2 Predicts the Infiltration of Immune Cells Into PC Microenvironment

**Figure 6:**
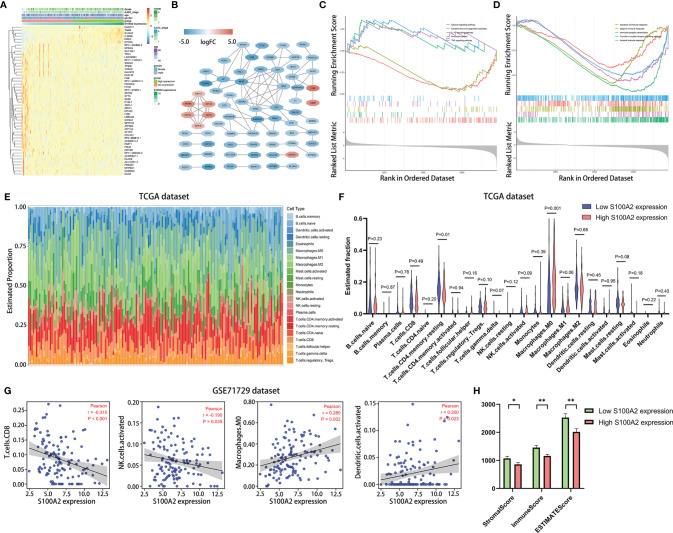
Differential gene analysis, co-expression network construction and functional enrichments analysis between S100A2 high and low expression groups, as well as the correlation analysis of the expression of S100A2 and immune cell infiltration. **(A)** Heatmap of top 50 DEGs in PC between S100A2 high and low expression groups. **(B)** Co-expression network of DEGs constructed and visualized with STRING database and Cytoscape. **(C, D)** Gene Set Enrichment Analysis between S100A2 high and low expression groups. The representative 5 KEGG enrichments **(C)** and GO enrichments **(D) **were displayed respectively. **(E)** The abundance ratio of the 22 types of immune cells in TCGA entire set. **(F) **Differential immune cell type abundance between S100A2 high and low expression groups. **(G)** Correlation analysis between the expression of S100A2 and the proportion of immune cells in GSE71729 dataset. Immune cell types with P < 0.05 were shown. **(H)** Differences in immune scores between high and low S100A2 expression groups. *P < 0.05; **P < 0.01.

Next, in order to investigate the in-depth mechanism of S100A2 leading to poor prognosis of PC, DEGs analysis was performed between the S100A2 high expression group (n = 83) and S100A2 low expression group (n = 83) in TCGA entire set (**Figure 6A**). As predicted, S100A2 was the gene with the most significant difference between the two groups, supporting the accuracy of the analysis. Then the co-expression network was constructed and visualized with STRING database and Cytoscape (**Figure 6B**).

To further elucidate the mechanism of S100A2, GSEA analysis was conducted on DEGs, in which P <0.05 and q <0.25 was considered statistically significant. Five representative pathways for the Kyoto Encyclopedia of Genes and Genomes (KEGG) and the Gene Ontology (GO) analyses were presented respectively (**Figures 6C, D**). Collectively, it was uncovered that part of the pathways of DEGs enrichment were associated with immune response and associated signaling pathways.

Therefore, CIBERSORT algorithm was applied to detect the proportions of 22 kinds of immune cells in TCGA entire set (**Figure 6E**). The results showed that relatively higher proportion of M0 macrophages cells and a lower proportion of resting memory CD4+ T cells were found in the S100A2 high expression group compared with the low expression group (**Figure 6F**). To further verify this conclusion, the number of macrophages and CD4+ T cells in the single cell dataset CRA001160 was statistically analyzed, which were divided into S100A2 high-expression group, S100A2 moderate-expression group and S100A2 low-expression group. Consistent with the previous results, with the increase of S100A2 expression, the proportion of macrophages gradually increased while that of CD4+T cells declined (**Figures S8A**–**C**) and immunohistochemical images also support these findings, in which patients with high S100A2 expression exhibited higher CD68 expression and lower CD4 expression (**Figures S8D, E**).

Moreover, in order to prove the universality of the results, GSE71729 dataset (n = 125) was also included for following analysis. It was discovered that the expression of S100A2 had a significant positive correlation with M0 macrophages and activated dendritic cells, while a remarkable negative correlation with CD8+ T cells and activated NK cells (**Figure 6G**). In addition, ESTIMATE package was also used to score the immune microenvironment, which revealed that the immune score of the group with high S100A2 expression was significantly lower than that of the group with low S100A2 expression (**Figure 6H**).

### S100A2 Is Associated With Patients’ TMB and Response to Immunotherapy

**Figure 7:**
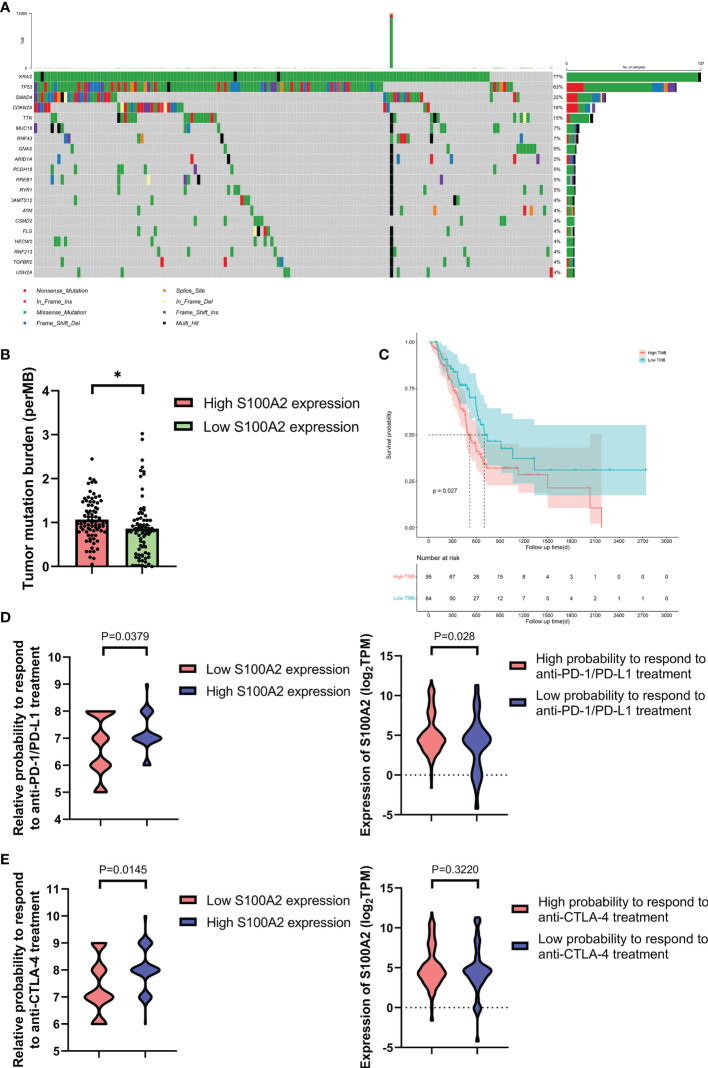
Figure 7. The mutation profile, TMB and relative probabilities to respond to immunotherapy in S100A2 high and low expression groups. **(A)** Mutation profile of PC patients in TCGA dataset. **(B)** The difference of TMB between S100A2 high and low expression groups. **(C)** Kaplan-Meier analysis of OS between the high TMB group and low TMB group. **(D, E)** The association between S100A2 expression and the relative probabilities to respond to immunotherapy, including anti-PD-1/PD-L1 therapy and anti-CTLA-4 therapy. *P < 0.05.

The mutation profiles of each PC patients were analyzed and visualized (**Figure S9**). For the TCGA dataset, the ten genes with the highest mutation rate were KRAS, TP53, SMAD4, CDKN2A, TTN, MUC16, RNF43, GNAS, ARID1A, and PCDH15 (**Figure 7A**). Meanwhile, we calculated the tumor mutation burden (TMB) of each sample and found that the TMB was higher in the group with high S100A2 expression (**Figure 7B**, P <0.05). Combined with the fact that patients with high TMB suffered a worse prognosis (**Figure 7C**, P <0.05), it was hypothesized that the effect of S100A2 on the progression of PC might result from a higher TMB.

IPS is a machine learning-based scoring system, which was able to predict patients’ response to immunotherapy including anti-PD-1/PD-L1 and anti-CTLA-4 treatment (37). Combined analysis of the expression S100A2 and IPS score proved that patients with high S100A2 expression had a relative high probability to respond to anti-PD-1/PD-L1 treatment and anti-CTLA-4 treatment (**Figures 7D, E**, P <0.05). These results indicated that patients with high S100A2 expression are more suitable for immunotherapy such as anti-PD-1/PD-L1 treatment and anti-CTLA-4 treatment.

### The Expression of S100A2 Was Positively Correlated With PD-L1 in PC Cells

**Figure 8:**
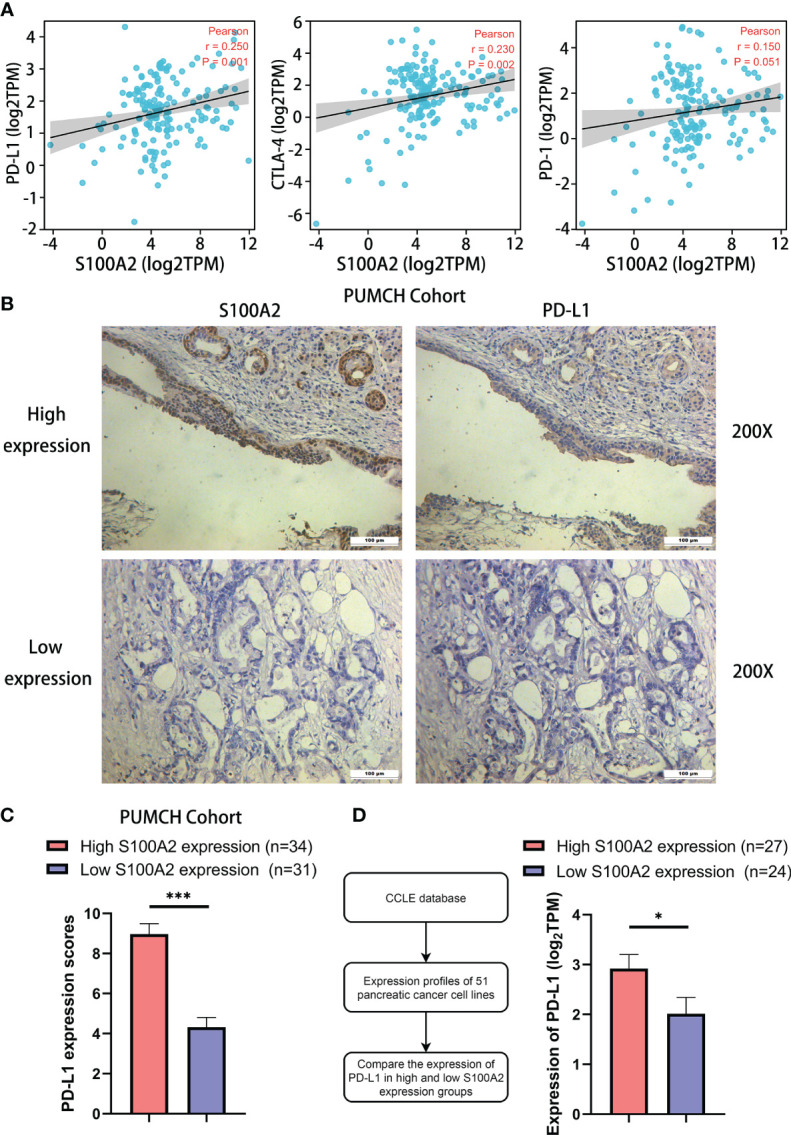
Correlation between S100A2 expression and PD-L1 expression in PC. **(A)** Correlation analysis between the expression of S100A2 and immune checkpoint, including PD-L1, PD-1 and CTLA-4. **(B)** Representative images of positive correlation between S100A2 and PD-L1 expression in sequential sections of PUMCH cohort. **(C)** Expression difference of PD-L1 in high and low S100A2 expression groups in PUMCH cohort (n = 65). **(D)** Expression difference of PD-L1 in pancreatic cancer cell lines with high and low S100A2 expression. *P < 0.05; ***P < 0.001.

In addition, it was discovered the expression of S100A2 in tumor tissues was remarkably positively correlated with the expression of PD-L1 (**Figure 8A**, P = 0.001, r = 0.25) and CTLA-4 (**Figure 8A**, P <0.01, r = 0.23), especially PD-L1. It might partly explain why samples with high expression of S100A2 experienced fewer CD8+ and CD4+ T cell infiltration, as well as better therapeutic effect on anti-PD1/PD-L1 therapy and anti-CTLA-4 therapy.

Since the relationship between S100A2 and PD-L1 was the most remarkable, PUMCH cohort (n = 65) was used to further demonstrate the positive correlation between S100A2 and PD-L1 (**Figure 8B** and **Table 3**). There was a significantly increased expression of PD-L1 in patients with high expression of S100A2 according to the immunohistochemical analysis of sequential sections staining S100A2 and PD-L1 (**Figure 8C**, P <0.001). Meanwhile, the expression profiles of 51 pancreatic cancer cell lines in Cancer Cell Line Encyclopedia (CCLE) database also supported above results (**Figure 8D**, P <0.05).

## Discussion

PC is one of the leading causes of cancer-related death worldwide, which is expected to become the second most common cause of cancer-related death by 2030 after lung cancer (40). There are a number of crucial reasons for this dismal status, and one of them is the lack of effective risk prediction models and biomarkers, which hinders individualized treatment of PC. Herein, due to the critical role of tumor microenvironment in the carcinogenesis and progression of PC (41, 42), we explored an IRGs-based predictive model to evaluate the prognosis of PC patients. Nine prognosis-specific IRGs were identified by a series of bioinformatics analysis: *S100A2*, *AREG*, *CXCL10*, *MET*, *OAS1*, *PI3*, *PLAU*, *S100A14*, and *SPP1*. Among them, AREG, CXCL10, MET, PLAU, S100A14, and SPP1 have been reported to be involved in the carcinogenesis and progression of PC (43–48), implying that our risk signature has considerable prognostic value. The remaining three genes, including S100A2, OAS1, and PI3, have not been well documented for their participation in PC development. Since S100A2 occupied the most paramount position in the risk signature-based DEGs analysis, and S100A2 accounted for a relatively high proportion in the risk signature, we tended to consider that S100A2 occupied the core position in the risk signature. Therefore, we gave special attention to S100A2 in the following exploration.

S100A2 is an important member of the S100 protein family, which is a group of highly conserved elongation factor (EF)-hand calcium-binding proteins (49, 50). Aberrant expression of S100A2 affects a range of cellular physiological functions, such as calcium homeostasis, enzyme activities and protein phosphorylation (51, 52). Notably, the role of S100A2 in tumors appears to be dual (53). Li et al. have reported that S100A2 activated the PI3K/AKT signaling pathway and upregulated GLUT1 expression in colorectal cancer, which induced glycolytic reprogramming and consequently increased tumor proliferation (54). Conversely, S100A2 was also identified to be one of the crucial tumor suppressor genes involved in the lung carcinogenesis (55). And our results supported its deteriorating effect in PC development. Previous clinical studies have proved S100A2 to be an independent poor prognostic factor and an indicator of less benefit to pancreatectomy for PC (56, 57). However, the underlying mechanism by which S100A2 promotes the progression of PC has not been fully revealed, which is also the main content of this study, especially the relationship between S100A2 and the tumor immune microenvironment. GSEA analysis revealed that the high expression of S100A2 was closely associated with the tumor immune microenvironment and corresponding pathways, enhanced interleukin-17 (IL-17), and tumor necrosis factor (TNF) signaling pathways, and also weakened adaptive immune response, which have been widely reported to participate in tumor progression (58–62).

Therefore, CIBERSORT algorithm was applied to further elucidate the abundance ratios of 22 types of immune cells in each PC patients from TCGA entire set. It was found that compared with S100A2 low expression patients, S100A2 high expression patients experienced significantly higher proportions of M0 macrophages cells and activated dendritic cells, as well as remarkable lower proportions of CD8+ T cells, resting memory CD4+ T cells and activated NK cells. Among them, CT8+T cells, the immune cell with the most prominent tumor killing ability (63, 64), were significantly reduced in S100A2 high expression group, which partially explained the poor prognosis of patients with high S100A2 expression. Meanwhile, NK cells, another major tumor killer cells (65, 66), showed a similar trend in S100A2 high expression group. In addition, M0 macrophages have been demonstrated to be associated with worse prognosis of PC (67), but some other researches reached the opposite conclusion (68). In our analysis, the high expression of S100A2 was associated with the increase of M0 macrophages, but whether this is related to the mechanism of S100A2 leading to PC progression remained to be explored. It was also possible that the increase of M0 macrophages is a precursor to the increase of tumor-associated macrophages (TAMs), and the immune profiles reflected in the TCGA and GSE71729 datasets were both prior to the differentiation of M0 macrophages. In addition, it was worth noting that the expression of S100A2 was positively correlated with the activation of dendritic cells, which played a pivotal role in anti-tumor immunity (69). For this phenomenon, we suspected that it might be due to the negative feedback effect caused by the decrease and functional deficiency of T cells.

In recent years, immunotherapy has been proved to be one of the most promising therapies for cancer therapy and has made a profound progress in prolonging the survival time of patients with of various types of tumors (70, 71). However, the immunotherapy is almost ineffective for pancreatic cancer (72, 73). Promisingly, a small subset of patients who exhibited high effector T-cell infiltration in tumor had longer overall survival (15, 16), implying that immunotherapy still had certain application value for PC patients.

Since we have previously explored the role of S100A2 in predicting tumor immune microenvironment, we wondered whether S100A2 has any predictive effect in predicting the efficacy of immunotherapy for PC. In the past years, studies have revealed that tumor mutation burden is positively related to the efficacy of immunotherapy (74, 75). Specifically, the more TMB a tumor has, the more neoantigens it is also likely to form and T-cells released by immune checkpoint inhibitors are more likely to recognize the neoantigens and thus attack the tumor cell. Therefore, we explored the relationship between the expression level of S100A2 and TMB. The results showed that patients with high S100A2 expression had higher TMB, which indirectly indicated that patients with high S100A2 expression might have better therapeutic effect on immunotherapy. Apart from that, according to the IPS algorithm (37), it was estimated that patients with high expression of S100A2 displayed relatively significant anti-PD1/PD-L1 and anti-CTLA-4 therapeutic effects. Moreover, the expression of S100A2 was remarkably positively correlated with the expression of PD-L1 and CTLA-4, especially with the expression of PD-L1. It has been reported that PD-L1 was able to inhibit the activation of T cells by binding to PD-1 receptor on the surface of T cells (76). In our study, we found that the expression of PD-L1 was significantly increased in patients with high S100A2 expression, suggesting that patients with high S100A2 expression may have fewer T cells infiltration in tumor microenvironment. Meanwhile, the results obtained by CIBERSORT algorithm also showed that patients with high S100A2 expression had fewer CD8+ T cells, which was exactly consistent with the previous speculation. To further verify the correlation between the expression of S100A2 and PD-L1, immunohistochemistry was performed on sequential sections of PUMCH cohort (n = 65) for S100A2 and PD-L1 respectively. According to comprehensive analysis of immunohistochemical scores, it was confirmed that patients with high S100A2 expression had higher PD-L1 expression in tumor tissues. In addition, expression profile of S100A2 and PD-L1 in all pancreatic cell lines was integrated from the CCLE database, and similar results were obtained. Regarding the co-expression of S100A2 and PD-L1, studies have shown that overexpression of S100A2 in A549 lung cancer cells enhanced Akt phosphorylation (77). Meanwhile, numerous studies have revealed that Akt activation could increase the expression of PD-L1 (78, 79). On this basis, we hypothesized that the co-expression of S100A2 and PD-L1 in pancreatic cancer might be based on the activation of the S100A2-Akt-PD-L1 signaling pathway.

In spite of the positive results, several limitations in our study should also be acknowledged. Firstly, due to the extremely poor prognosis of PC, the survival time of patients rarely exceeds three years, which may bring some imprecise results when we want to predict long-term prognosis. Besides, IPS algorithm is applied to mimic patients’ response to immunotherapy. Although the prediction of immunotherapy efficacy by IPS algorithm has been verified in several independent datasets, it still cannot completely replace the actual therapeutic effect.

In summary, a risk signature consisting of nine immune-related genes was constructed through a series of bioinformatics analysis, which was validated in TCGA training set, TCGA testing set, TCGA entire set, GSE78229 set and GSE62452 set. Subsequently, a nomogram was also developed to establish a more accurate prognostic prediction model for PC. Furthermore, S100A2 was identified as the gene occupying the core position in risk model, which was demonstrated to be significantly associated with the progression of tumor grade, AJCC_stage, age and T stage. Mechanically, GSEA, ESTIMATE and CIBERSORT algorithm analysis revealed that the deteriorating effect of S100A2 was associated with dysfunctional tumor immune microenvironment, mainly related to lower proportion of CD8+T cells and activated NK cells and higher proportion of M0 macrophages. Meanwhile, the results of IPS algorithm revealed that patients with high expression of S100A2 might get more benefit from immunotherapy. Finally, our independent cohort was applied to demonstrate a remarkably positive correlation between the expression of S100A2 and PD-L1, as well as the positive relationship between S100A2 expression and unfavorable prognosis of PC patients. Our findings demonstrate S100A2 might be responsible for the preservation of immune-suppressive status in PC microenvironment, which contributes to accurate assessment of the prognosis of PC patients and optimization of the clinical decision-making.

## Data Availability Statement

The original contributions presented in the study are included in the article/**Supplementary Material**. Further inquiries can be directed to the corresponding author.

## Ethics Statement

Written informed consent was obtained from all the patients enrolled in this study. This project was approved by the Ethics Committee of Peking Union Medical College Hospital.

## Author Contributions

Study concept and design: YC, CW, JS, RX, RR, and YZ. Experimental design and implementation: YC and CW. Drafting of the manuscript: YC. Critical revision of the manuscript for important intellectual content: YC, CW, JS, RX, RR, and YZ. Obtained funding: YZ. All authors contributed to the article and approved the submitted version.

## Funding

This study was supported by the CAMS Innovation Fund For Medical Sciences (2021, 2021-1-I2M-002, to YZ), National Nature Science Foundation of China (2021, 82102810, to CW) and fellowship of China Postdoctoral Science Foundation (2021, 2021M700501, to CW).

## Conflict of Interest

The authors declare that the research was conducted in the absence of any commercial or financial relationships that could be construed as a potential conflict of interest.

## Publisher’s Note

All claims expressed in this article are solely those of the authors and do not necessarily represent those of their affiliated organizations, or those of the publisher, the editors and the reviewers. Any product that may be evaluated in this article, or claim that may be made by its manufacturer, is not guaranteed or endorsed by the publisher.

## Supplementary Material

Supplementary Figure 1 Flowchart of the whole study.

Supplementary Figure 2 The copy number variation information of the nine hub IRGs in TCGA dataset.

Supplementary Figure 3 Representative images of hub IRGs expression status except CXCL10 in normal pancreas and PC tissues. (The expression information of CXCL10 was unavailable in HPA database)

Supplementary Figure 4 Independence of the risk signature and the other clinical variables, including gender, age, AJCC_stage and grade. **(A, C, E)** Univariate Cox regression analyses in the training set, testing set and entire set. **(B, D, F)** Multivariate Cox regression analyses in the training set testing set and entire set.

Supplementary Figure 5 Stratification analyses of all patients using the risk signature. **(A–C)** The Kaplan-Meier analysis of the younger stratum (age ≤ 65, n=88), older stratum (age >65, n=78) and all patients with PC (n=166). **(D–F)** The Kaplan-Meier analysis of the male stratum (n=90), female stratum n=76) and all patients with PC (n=166). **(G–I)** The Kaplan-Meier analysis of the Grade I/II stratum (n=117), Grade III/IV stratum (n=49) and all patients with PC (n=166). **(J-L)** The Kaplan-Meier analysis of the T1+T2 stratum (n=27), T3+T4 stratum (n=139) and all patients with PC (n=166).

Supplementary Figure 6 Validation of the nomogram in training set, testing set and entire set. **(A–I)** The calibration plot of the nomogram for agreement test between 1-, 1.5- and 2-year OS prediction and actual outcome in the training set, testing set and entire set.

Supplementary Figure 7 A pan-cancer analysis of S100A2 on 33 types of tumors. Red represented a significant increase in tumor, green represented a significant decrease in tumor, and black meant no significant change.

Supplementary Figure 8 The change of macrophages and CD4+ T cells with the increase of S100A2 expression. **(A)** The expression of S100A2 in cancer cells from 24 PDAC patients in single cell dataset CRA001160 (From high to low). **(B, C)** The proportion of macrophages and CD4+ T cells in high S100A2 expression group, moderate S100A2 expression group and low S100A2 expression group. **(D, E)** Representative images of S100A2 and CD68/CD4 expression by immunohistochemistry in HPA database.

Supplementary Figure 9 The mutation profiles of patients in TCGA dataset.

Supplementary Table 1 Detailed clinical and pathologic information of TCGA entire set.

Supplementary Table 2 Detailed clinical and pathologic information of GSE62452 dataset.

Supplementary Table 3 Detailed clinical and pathologic information of GSE78229 dataset.

Supplementary Table 4 Immune-related genes obtained from the ImmPort database.

Supplementary Table 5 Prognosis-related genes obtained by differential expressed gene analysis and univariate Cox regression analysis.
