# Supplementary figures and images for: S100A2 Is a Prognostic Biomarker Involved in Immune Infiltration and Predict Immunotherapy Response in Pancreatic Cancer
Source: Front Immunol. 2021 Nov 23;12:758004. doi: 10.3389/fimmu.2021.758004 (PMC8650155; doi:10.3389/fimmu.2021.758004)

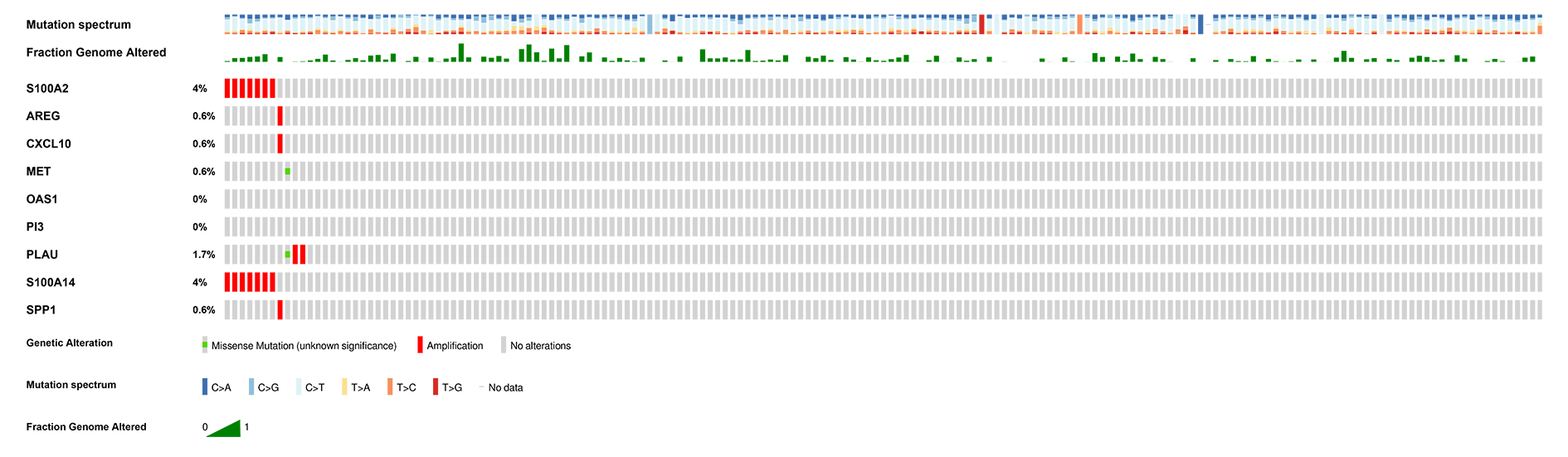

Supplement: Supplementary Figure 1 — Flowchart of the whole study. [file DataSheet_1.zip › Figure S2.tif]

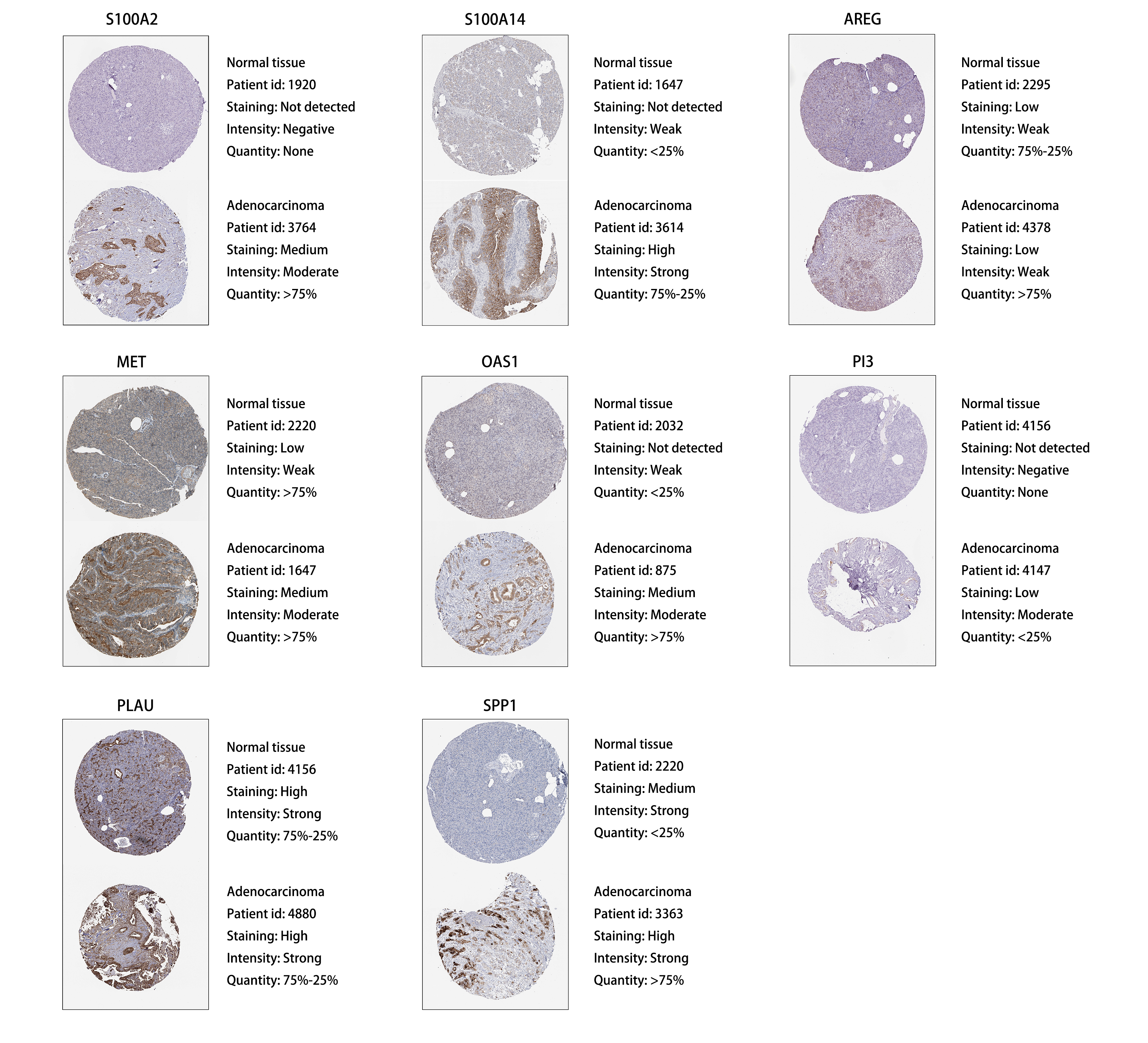

Supplement: Supplementary Figure 1 — Flowchart of the whole study. [file DataSheet_1.zip › Figure S3.tif]

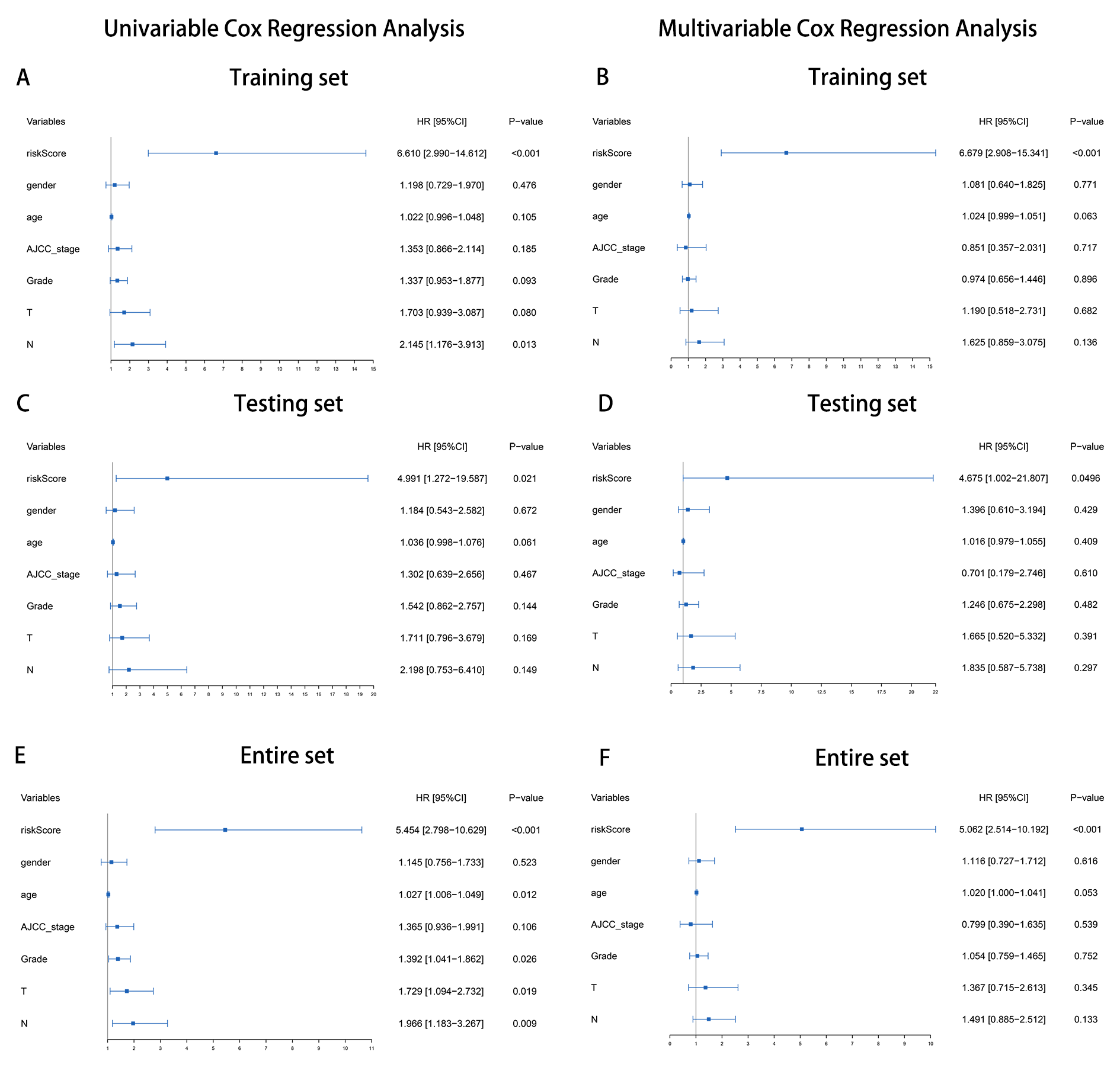

Supplement: Supplementary Figure 1 — Flowchart of the whole study. [file DataSheet_1.zip › Figure S4.tif]

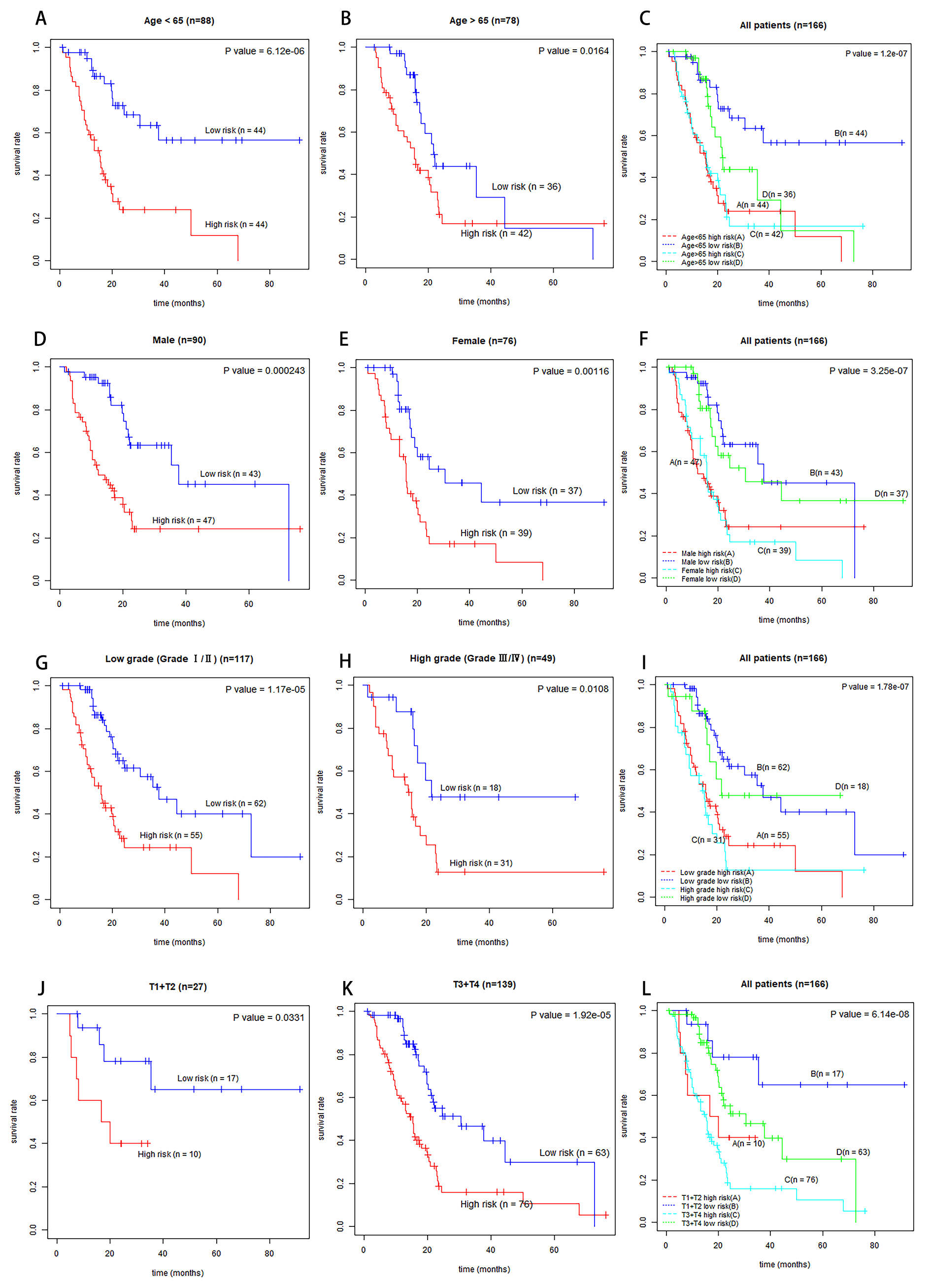

Supplement: Supplementary Figure 1 — Flowchart of the whole study. [file DataSheet_1.zip › Figure S5.tif]

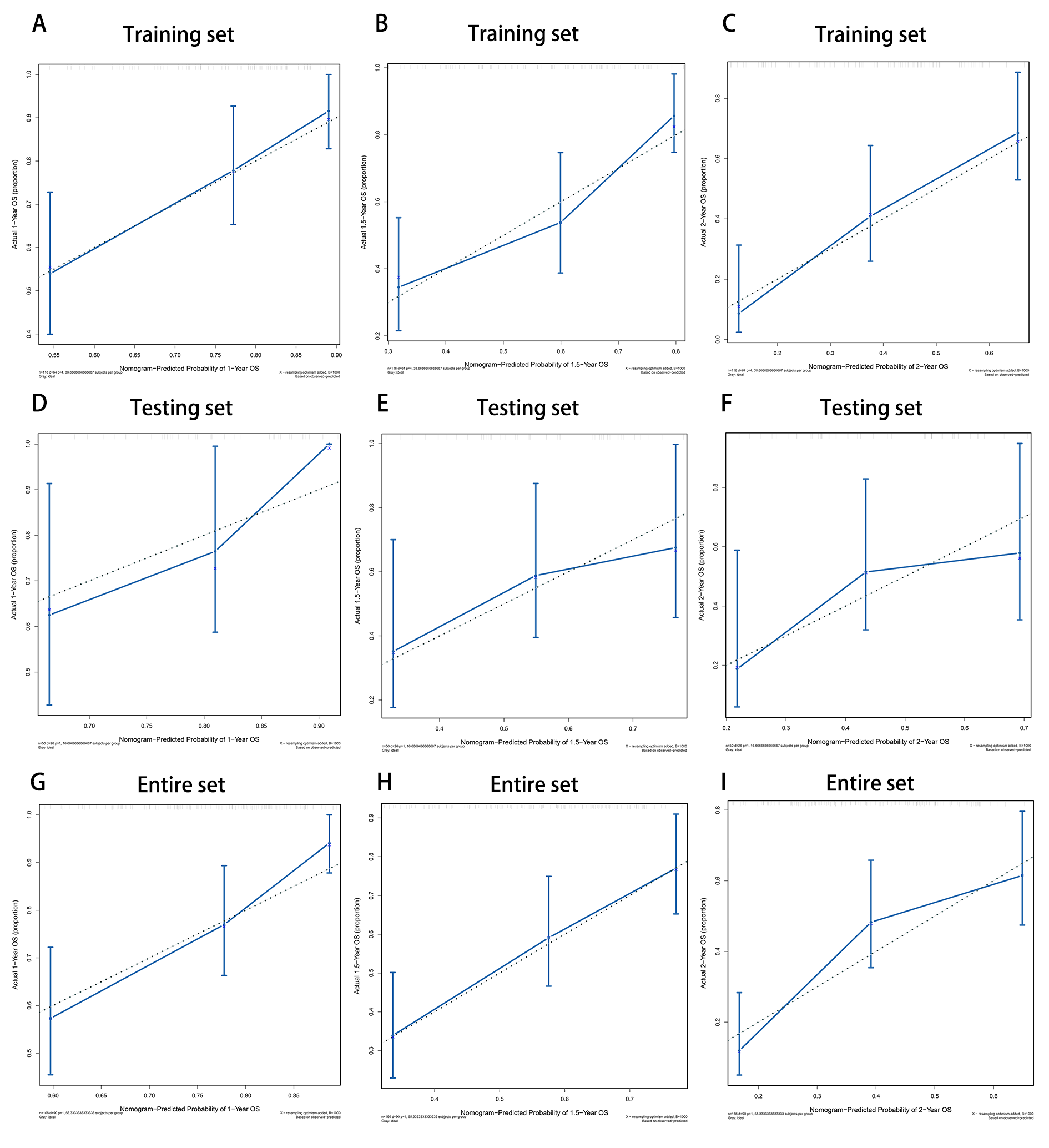

Supplement: Supplementary Figure 1 — Flowchart of the whole study. [file DataSheet_1.zip › Figure S6.tif]

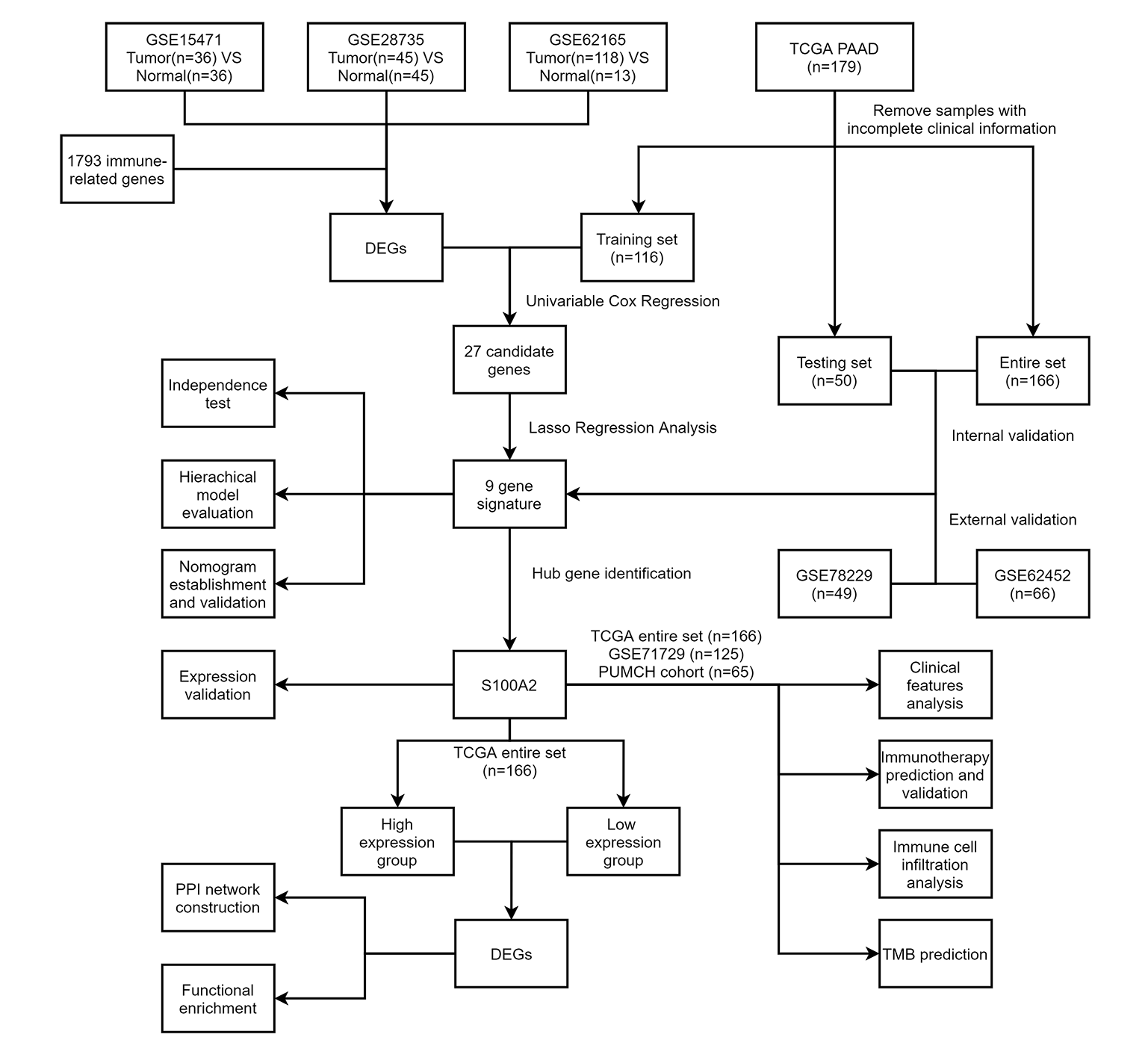

Supplement: Supplementary Figure 1 — Flowchart of the whole study. [file DataSheet_1.zip › Figure S1.tif]

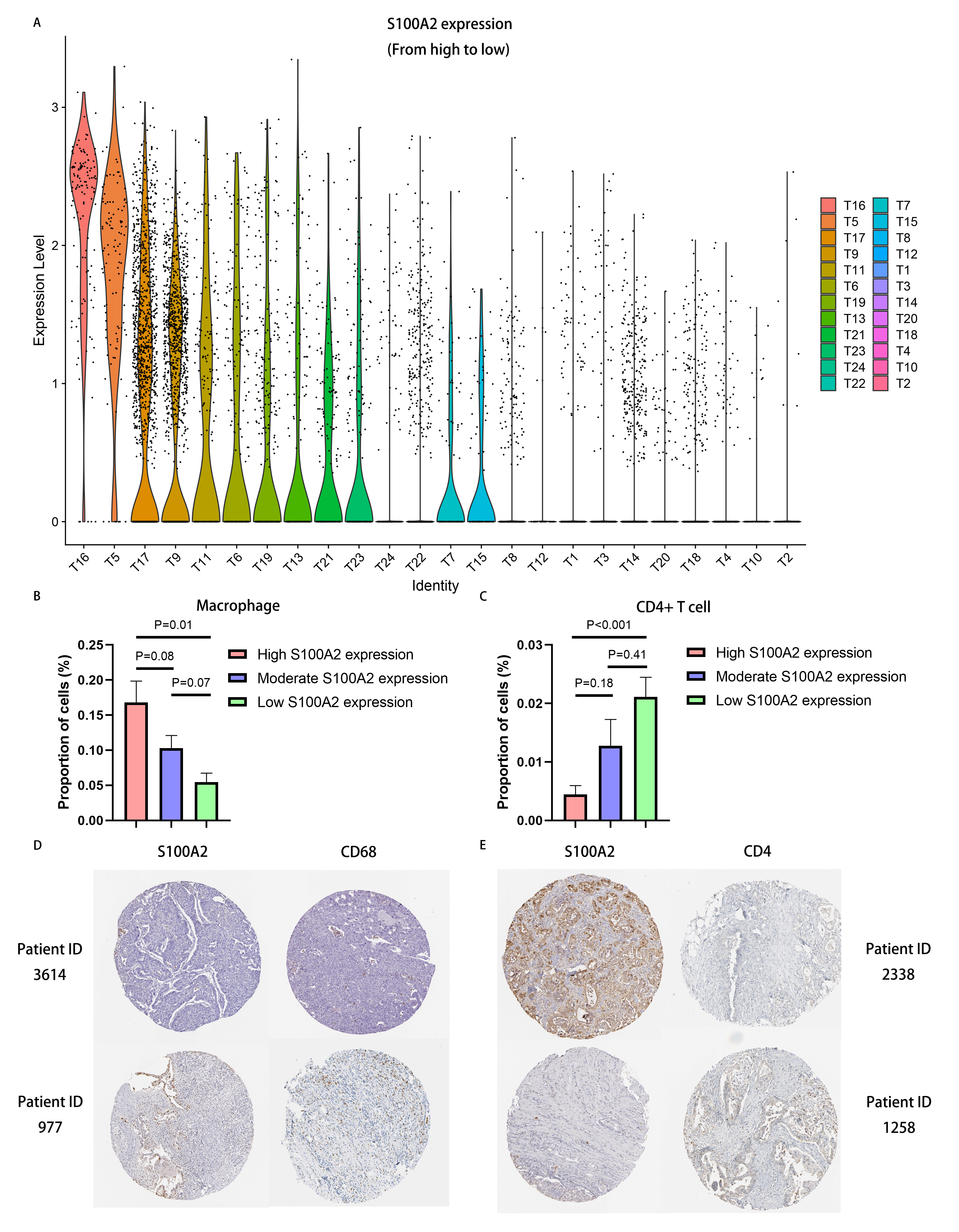

Supplement: Supplementary Figure 7 — A pan-cancer analysis of S100A2 on 33 types of tumors. Red represented a significant increase in tumor, green represented a significant decrease in tumor, and black meant no significant change. [file DataSheet_2.zip › Figure S8.tif]

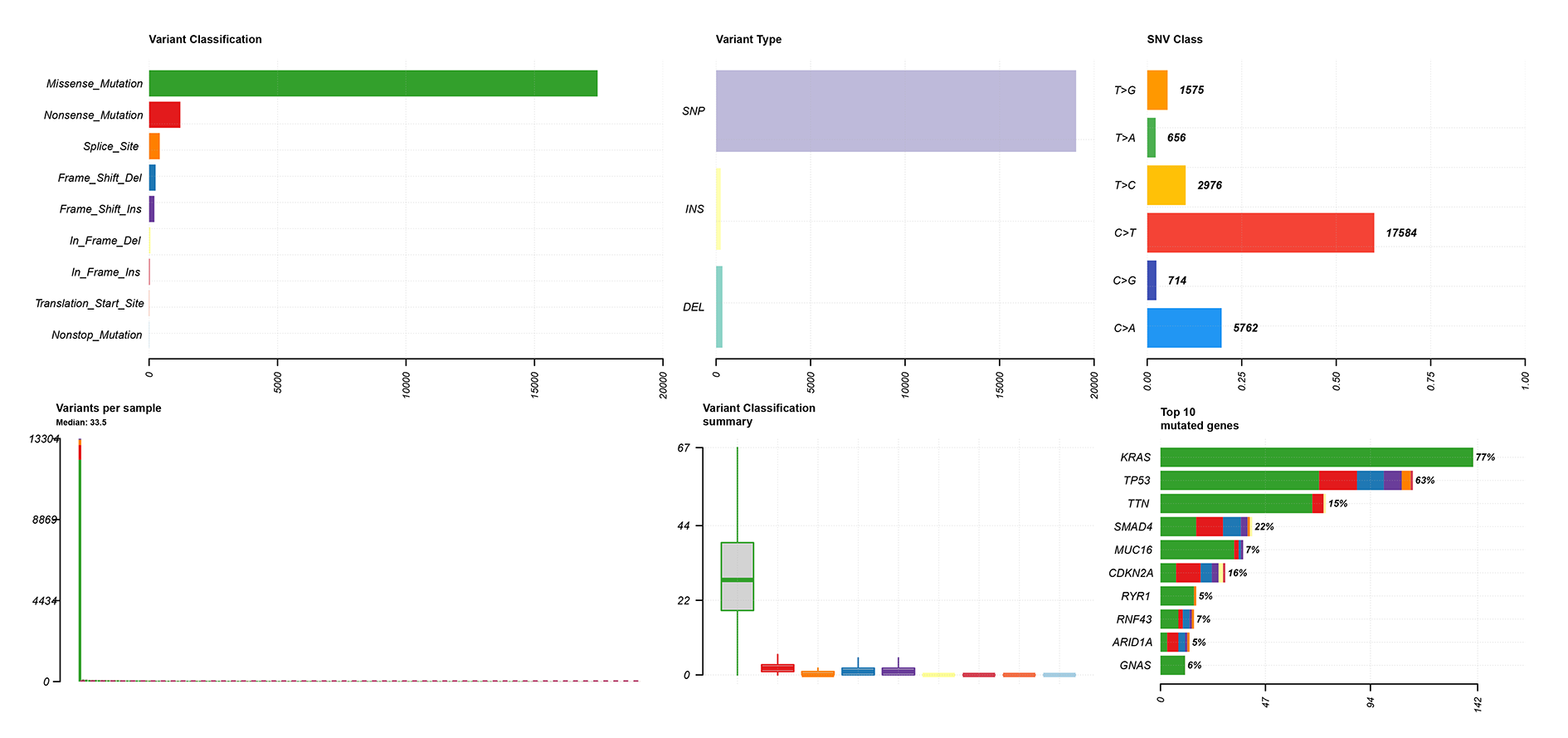

Supplement: Supplementary Figure 7 — A pan-cancer analysis of S100A2 on 33 types of tumors. Red represented a significant increase in tumor, green represented a significant decrease in tumor, and black meant no significant change. [file DataSheet_2.zip › Figure S9.tif]

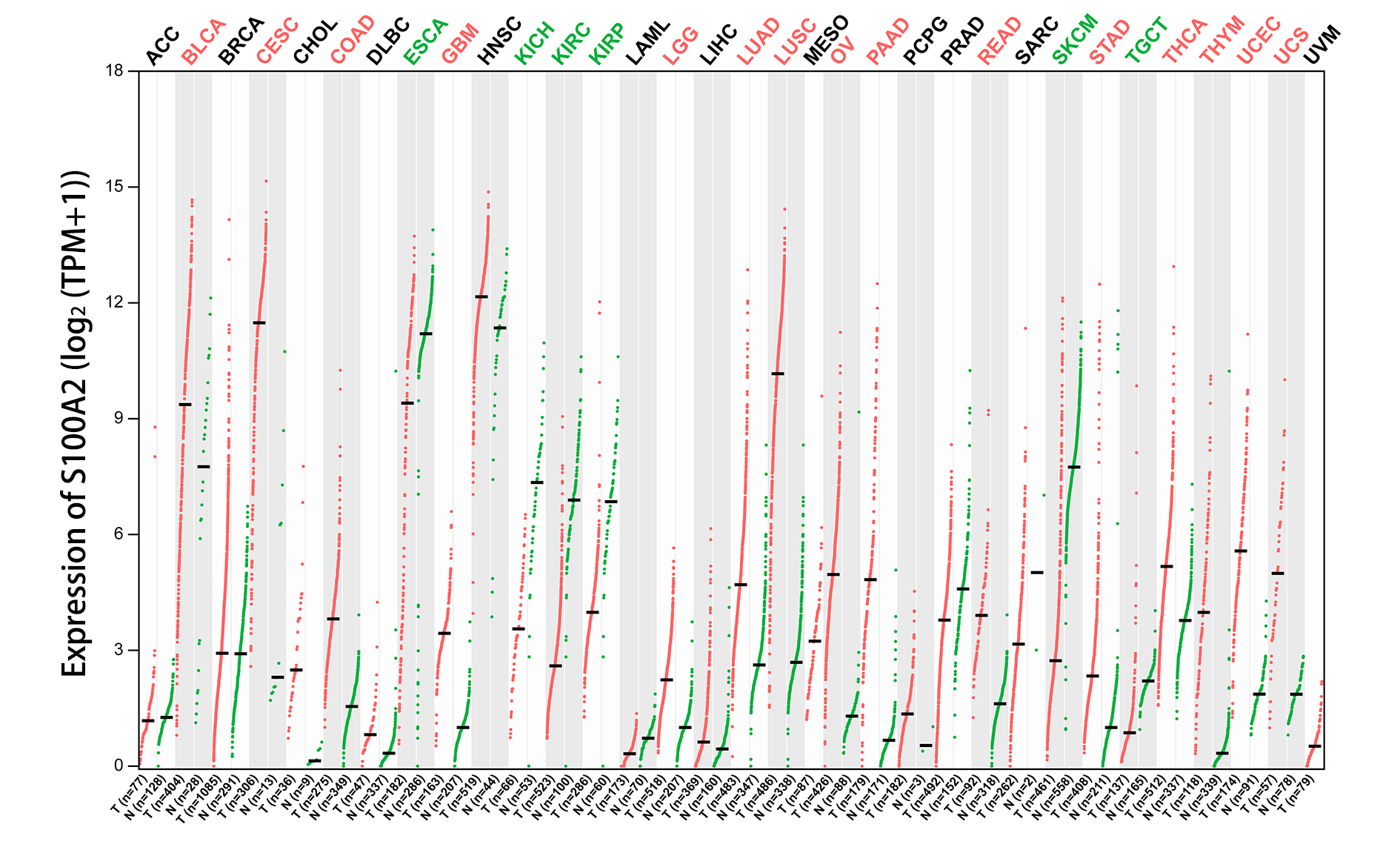

Supplement: Supplementary Figure 7 — A pan-cancer analysis of S100A2 on 33 types of tumors. Red represented a significant increase in tumor, green represented a significant decrease in tumor, and black meant no significant change. [file DataSheet_2.zip › Figure S7.tif]
